# 14-Deoxy-11,12-didehydroandrographolide Alleviates IL-1β-Induced Insulin Resistance by Modulating NOX2-Driven ROS Generation and Restoring Insulin Signaling in 3T3-L1 Adipocytes

**DOI:** 10.3390/antiox14101155

**Published:** 2025-09-24

**Authors:** Chih-Ching Yen, Chia-Wen Lo, Jyun-Lin Lee, Kai-Li Liu, Chien-Chun Li, Chong-Kuei Lii, Chia-En Hsu, Ya-Chen Yang, Haw-Wen Chen

**Affiliations:** 1 Department of Internal Medicine, China Medical University Hospital, Taichung 404328, Taiwan; 005210@tool.caaumed.org.tw; 2 Department of Nutrition, College of Medical and Health Care, Hung-Kuang University, Taichung 433304, Taiwan; cook0912@sunrise.hk.edu.tw; 3 School of Chinese Medicine, College of Chinese Medicine, China Medical University, Taichung 404328, Taiwan; jyunlinlee@mail.cmu.edu.tw; 4 Department of Nutrition, Chung Shan Medical University, Taichung 402306, Taiwan; kaililiu@csmu.edu.tw (K.-L.L.); licc@csmu.edu.tw (C.-C.L.); 5 Department of Nutrition, Chung Shan Medical University Hospital, Taichung 402306, Taiwan; 6 Department of Nutrition, China Medical University, No. 100, Section 1, Jingmao Road, Beitun District, Taichung 406040, Taiwan; cklii@mail.cmu.edu.tw (C.-K.L.); cshc266@csh.org.tw (C.-E.H.); 7 Department of Health and Nutrition Biotechnology, Asia University, Taichung 413305, Taiwan; yachenyang@asia.edu.tw

**Keywords:** 14-Deoxy-11,12-didehydroandrographolide (deAND), IL-1β, insulin resistance (IR), network pharmacology, reactive oxygen species (ROS), 3T3-L1 adipocytes

## Abstract

Obesity is closely associated with the development of insulin resistance (IR) and type 2 diabetes mellitus (T2DM), primarily due to dysfunctional adipose tissue expansion and the secretion of pro-inflammatory cytokines such as interleukin-1β (IL-1β). 14-Deoxy-11,12-didehydroandrographolide (deAND), a major diterpenoid component of Andrographis paniculata, has demonstrated notable antioxidant and anti-inflammatory activities. This study aimed to investigate the protective effects and mechanisms of deAND against IL-1β-induced IR in 3T3-L1 adipocytes. Network pharmacology analysis indicated that deAND targets several IR-related signaling pathways, particularly the MAPK and IRS-1/AKT pathways. The experimental results show that IL-1β stimulated p67phox membrane translocation and reactive oxygen species (ROS) production, contributing to impaired insulin signaling by activating ERK and JNK and reducing IRS-1/AKT phosphorylation, which ultimately decreased insulin-stimulated glucose uptake. Pretreatment with deAND effectively inhibited NOX2-derived ROS generation, suppressed ERK/JNK activation, restored IRS-1/AKT phosphorylation, and reversed the reduction in glucose uptake caused by IL-1β. These findings suggest that deAND can alleviate IR by inhibiting NOX2-mediated oxidative stress, restoring insulin signaling and improving glucose uptake, highlighting its potential as a therapeutic agent for obesity-related IR.

## 1. Introduction

Obesity is becoming a challenging public health issue for government agencies throughout the world [1]. Obesity is linked to inflammation, oxidative stress, and hyperlipidemia, which are key factors in the development of insulin resistance (IR) and type 2 diabetes mellitus (T2DM) [2]. Inflammation is a critical contributor to adipose tissue dysfunction [3,4], recruiting immune cells that secrete pro-inflammatory cytokines like tumor necrosis factor α (TNFα), interleukin 1β (IL-1β), and IL-6 [5,6]. Compared with an increase in IL-6 level alone, a combined increase in both IL-1β and IL-6 is associated with a greater risk of developing T2DM [7]. Notably, XOMA 052, an anti-IL-1β monoclonal antibody, has been demonstrated to prevent IL-1β-mediated IR in 3T3-L1 adipocytes [8]. Therefore, IL-1β signaling may play an important role in IR in adipocytes.

Oxidative stress, defined as an imbalance between reactive oxygen species (ROS) production and clearance, also plays a role in IR formation [9]. NADPH oxidases (NOX), major sources of cellular ROS, transfer electrons across the plasma membrane to molecular oxygen, generating superoxide anions [10]. NOX2, expressed in various immune cells and adipocytes, is particularly significant [11]. Studies have shown that NOX2 knockout mice accumulate less body fat and have improved IR after being fed a high-fat diet (HFD), highlighting the role of NOX2 in increasing adiposity and blood glucose disturbances associated with HFD consumption [11].

Insulin signaling is impaired in IR. Normally, insulin binds to the insulin receptor, inducing tyrosine autophosphorylation of the β-subunit of receptor [12]. This activation leads to phosphorylation of insulin receptor substrates (IRS) 1 and 2, which then phosphorylate downstream signaling mediators such as PI3K, PDK1, AKT, and AS160, facilitating GLUT4 translocation to the cell membrane and promoting glucose uptake [13]. However, in obesity-induced IR, aberrant IRS-1 phosphorylation impedes insulin signal transduction [14]. Research has shown that pro-inflammatory cytokines promote phosphorylation of IRS-1 at serine or threonine residues through various kinases, including ERK, JNK, and IκB kinase (IKK), which inhibits IRS-1 tyrosine phosphorylation in response to insulin [12].

Andrographis paniculata (Burm.f.) Nees, commonly known as A. paniculata, is a medicinal herb known for its wide array of physiological effects, including antioxidant [15], anti-inflammatory [16], hepato-protective [15], and antidiabetic properties [17]. Among its active compounds, andrographolide (AND) and dehydroandrographolide (deAND) are prominent diterpenoids [18]. Research has highlighted the pharmacological benefits of AND, such as anti-inflammatory, antioxidant, and anti-hyperglycemic activities [19]. However, there is limited exploration into the pharmacological role of deAND compared with AND. Due to its antioxidant and anti-inflammatory properties [16,20], our interest was in investigating whether deAND, a constituent of traditional Chinese medicine renowned for these qualities, could mitigate IL-1β-induced IR in 3T3-L1 adipocytes and elucidate the underlying mechanisms.

## 2. Materials and Methods

### 2.1. Reagents

DMEM (high glucose, 4.5 g/L), penicillin, and 0.25% trypsin-EDTA were obtained from Gibco (Grand Island, NY, USA). Cosmic calf serum was purchased from HyClone (Logan, UT, USA). Insulin was from Millipore (Billerica, MA, USA). Dexamethasone, 3-isobutyl-1-methylxanthine (IBMX), PD98059, SP600125, Bay11-7082, and IL-1β were from Sigma-Aldrich (St. Louis, MO, USA). Antibodies against p-AKT (Thr308), p-IRS-1 (Ser307), p-JNK, p-ERK, p-IKK, p67phox, and Na+-K+ ATPase were obtained from Cell Signaling (Boston, MA, USA); p-IRS-1 (Tyr608) and the secondary antibody from Millipore (Billerica, MA, USA); and α-actin were from Sigma-Aldrich (St. Louis, MO, USA). 2′,7′-Dichlorodihydrofluorescein diacetate (DCFDA) was purchased from Invitrogen (Carlsbad, CA, USA).

### 2.2. Preparation of deAND

deAND was purified from *A. paniculata* using gel filtration and recrystallization, as previously described [16]. Briefly, dried *A. paniculata* was powdered and extracted with 95% ethanol (1:5, *w*/*v*). The filtrate was collected and concentrated using a rotary evaporator (N1000V, EYELA). The resulting concentrate was then extracted with ethyl acetate (EA)/H_2_O (1:1, *v*/*v*), and the EA layer was mixed with an equal volume of silica gel (70–230 mesh) for adsorption. After evaporating the EA, the silica gel containing the adsorbed compounds was layered on top of a column filled with un-adsorbed silica gel. The column was sequentially eluted with the following solvents: n-hexane (n-H)/EA (10:1), n-H/EA (5:1), n-H/EA (3:1), n-H/EA (1:1), n-H/EA (1:2), n-H/EA (1:5), EA, EA/methanol (10:1), and EA/methanol (5:1). Fractions containing deAND were identified by thin-layer chromatography, pooled, and allowed to crystallize by evaporating the solvent in a fume hood. The identity of deAND was confirmed by LC-MS, and its purity was approximately 97.9% as determined by HPLC analysis.

### 2.3. Cell Culture and Differentiation

3T3-L1 preadipocytes were obtained from Bioresources Collection and Research Center (BCRC, Hsin-Chu, Taiwan). 3T3-L1 preadipocytes were cultured in a maintenance medium, and differentiation media I and II were used to induce mature adipocytes, as described previously [21].

### 2.4. Target Prediction

**Figure 1:**
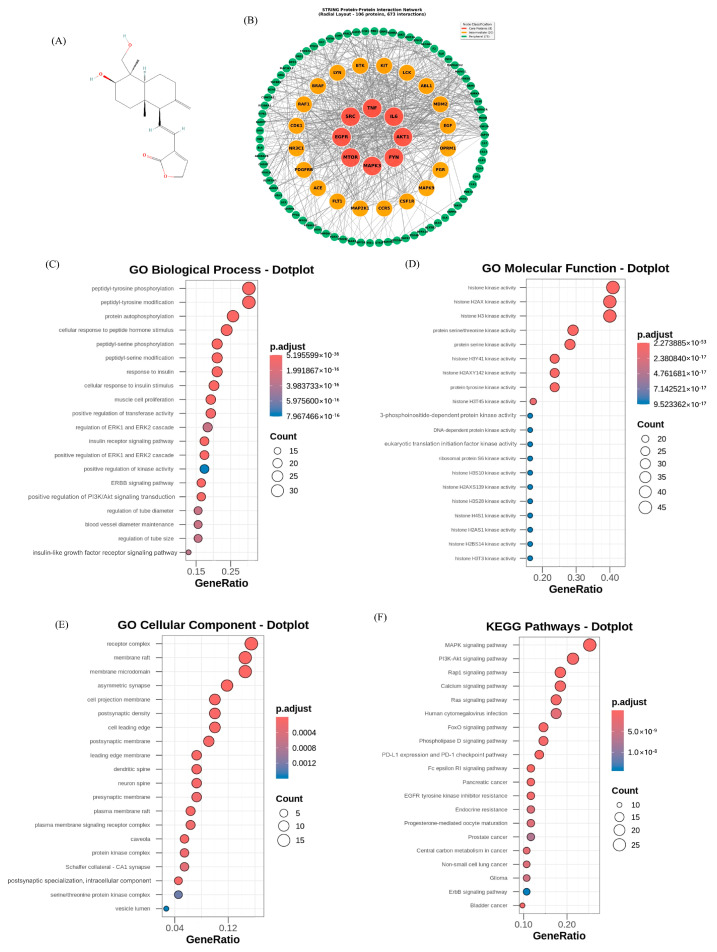
Network pharmacology of deAND and IR. (**A**) The 2D structure of deAND. (**B**) PPI network of intersection genes. The node size and color reflect the number of combined targets (degree) and *p*-value, respectively. GO analysis: deAND-related (**C**) biological processes (**D**) molecular functions, and (**E**) cellular components. (**F**) KEGG pathway analysis.

The two-dimensional (2D) molecular structure of deAND (Figure 1A) was retrieved from the PubChem database (https://pubchem.ncbi.nlm.nih.gov, accessed on 9 June 2025). Potential drug targets of deAND were predicted using SwissTargetPrediction (http://www.swisstargetprediction.ch/, accessed on 9 June 2025) by uploading its 2D molecular structure.

### 2.5. PPI Network Construction and Analysis

The protein–protein interaction (PPI) network was constructed using the STRING database (https://string-db.org). Interaction data were retrieved via the STRING API with a minimum confidence score threshold of 0.3, which reflects the strength of functional associations. The resulting network was visualized using a customized radial layout implemented in Matplotlib (version 3.7). Based on calculated importance scores, proteins were classified into three groups: core, intermediate, and peripheral. The thickness and color intensity of the edges were adjusted according to the confidence values obtained from STRING.

### 2.6. GO and KEGG Enrichment Analysis

Potential protein targets of the compound were predicted using SwissTargetPrediction, restricted to mouse-specific targets. To investigate the biological relevance of these targets, functional enrichment analyses were conducted for Gene Ontology (GO) and Kyoto Encyclopedia of Genes and Genomes (KEGG) pathways using the clusterProfiler package. GO analysis was performed separately for the three domains—Biological Process (BP), Molecular Function (MF), and Cellular Component (CC)—with statistical significance defined by an adjusted *p*-value ≤ 0.05 and a *q*-value ≤ 0.20, based on the Benjamini–Hochberg correction. KEGG pathway analysis was conducted in parallel using the same significance thresholds. For both GO and KEGG results, the top 20 enriched terms, ranked by adjusted *p*-value, were selected for visualization.

### 2.7. Cell Viability Assay

Cell viability was evaluated using the MTT assay. Cells were grown to 70–80% confluence and pretreated with deAND (2.5, 5, 10, or 20 μM) for 16 h, followed by exposure to IL-1β (10 ng/mL) for 24 h. The assay was performed as previously described [22].

### 2.8. Oil Red O Staining

After each experiment, cells were washed twice with PBS and fixed with 10% formaldehyde for 10 min. They were then stained with 0.3% Oil Red O (#O0625, Sigma) for 5 min. Lipid accumulation was evaluated by bright-field microscopy, and lipid content was quantified by extracting the dye with isopropanol, followed by absorbance measurement at 510 nm using a BioTek Synergy HT microplate reader (Winooski, VT, USA).

### 2.9. Subcellular Fractionation

Membrane and cytosolic proteins were extracted using the Mem-PER™ Plus Membrane Protein Extraction Kit (Thermo Fisher Scientific, Waltham, MA, USA). Total protein concentrations were determined with the Coomassie Plus Protein Assay Kit (Bio-Rad, Hercules, CA, USA).

### 2.10. Western Blotting Analysis

After each experiment, cells were washed twice with ice-cold PBS and lysed in RIPA buffer. Lysates were centrifuged at 12,000× *g* for 30 min at 4 °C, and the supernatant was collected as total cellular protein. Protein concentrations were determined using the Coomassie Plus Protein Assay Kit (Bio-Rad, Hercules, CA, USA). For Western blot analysis, 10 μg of total protein was loaded per lane, separated on 8% SDS-polyacrylamide gels, and transferred to polyvinylidene difluoride membranes (Millipore). Membranes were blocked with 5% nonfat dry milk at room temperature for 1 h and then incubated overnight at 4 °C with primary antibodies: anti–p-IRS-1 (Tyr608) (Cat# 09-432, Millipore, St. Louis, MO, USA; 1:1000), anti–p-IRS-1 (Ser307) (Cat# 2381, Cell Signaling Technology, Danvers, MA, USA; 1:1000), anti–p-AKT (Thr308) (Cat# 4056, Cell Signaling Technology; 1:1000), anti–p-ERK1/2 (Cat# 9101, Cell Signaling Technology; 1:1000), anti–p-JNK (Cat# 4668, Cell Signaling Technology; 1:1000), anti–p-IKK (Cat# 2697, Cell Signaling Technology; 1:1000), anti–p67phox (Cat# 3923, Cell Signaling Technology; 1:1000), anti–Na^+^/K^+^-ATPase (Cat# 3010, Cell Signaling Technology; 1:1000), and anti–α-actin (Cat# A2066, Sigma-Aldrich, St. Louis, MO, USA; 1:5000). After washing, membranes were incubated for 1 h at room temperature with horseradish peroxidase (HRP)-conjugated secondary antibodies: anti-rabbit IgG-HRP (Cat# A0545, Sigma-Aldrich; 1:5000) or anti-mouse IgG-HRP (Cat# A9044, Sigma-Aldrich; 1:5000). Protein bands were detected using an enhanced chemiluminescence kit (Bio Kit, Miaoli, Taiwan) and imaged with a Fuji Film LAS-4000 luminescence analyzer (Tokyo, Japan). Band intensities were quantified using ImageJ software (version 1.54, NIH, Bethesda, MD, USA).

### 2.11. Glucose Uptake Assay

The fluorescent glucose analog 2-(N-(7-nitrobenz-2-oxa-1,3-diazol-4-yl)amino)-2-deoxyglucose (2-NBDG; Invitrogen, Leicestershire, UK) was used to assess glucose uptake in adipocytes. Differentiated 3T3-L1 cells were pretreated with deAND (5 or 10 μM) for 16 h, followed by IL-1β (10 ng/mL) for 23 h. After washing with cold PBS, cells were incubated in low-glucose, serum-free medium containing deAND and IL-1β for 1 h. Cells were then stimulated with 100 nM insulin and 600 μM 2-NBDG for 30 min. After two PBS washes, cells were fixed with 10% formalin, and fluorescence intensity was visualized using a fluorescence microscope and quantified using ImageJ software (NIH, USA).

### 2.12. Reactive Oxygen Species Measurement

Intracellular oxidative stress was measured using the DCFDA probe as previously described [23]. Cells were pretreated with deAND (5 or 10 μM) for 16 h, followed by incubation with IL-1β (10 ng/mL) and DCFDA (40 μg/mL) for 40 min. Cells were then fixed with 10% formalin to preserve morphology, and fluorescence intensity was assessed using a Revolve fluorescence microscope (Echo Laboratories, San Diego, CA, USA).

### 2.13. Statistical Analysis

Data were analyzed using one-way analysis of variance (ANOVA) with SAS software (version 9.4, SAS Institute, Cary, NC, USA). Differences among group means were further evaluated using Duncan’s multiple range test. Student’s t-test was applied to compare specific pairs of means. Statistical significance was defined as *p* < 0.05.

## 3. Results

### 3.1. Retrieval of Targets and Construction of PPI Network

deAND-binding proteins were predicted from the SwissTargetPrediction (http://www.swisstargetprediction.ch/, accessed on 9 June 2025) database (100 proteins). Genes associated with IR were obtained from the GeneCards, OMIM, and DisGeNET databases, as well as from the published literature, following the approach described by Liu et al. [24]. The top 10 hub genes identified were INS, AKT1, IL-6, TP53, TNF, VEGFA, MAPK3, EGFR, EGF, and SRC. Drug-related genes and disease-related genes were merged to obtain potential targets of deAND for IR treatment (10 proteins). Finally, 110 proteins (100 predicted by SwissTargetPrediction, listed in Supplementary Table S1, along with 10 potential targets for IR treatment) were used for further analysis. The PPI network established using the STRING database consisted of 106 nodes and 673 edges. Based on the STRING protein–protein interaction network analysis (Figure 1B), the top 10 targets ranked in order of degree were SRC, EGFR, AKT1, MTOR, TNF, IL6, MAPK3, FYN, LYN, and BTK. These targets may have critical roles in treatment.

### 3.2. GO and KEGG Pathway Enrichment

Based on the functional enrichment analysis, GO analysis yielded multiple biological processes, molecular functions, and cellular components. The main biological processes were peptidyl-tyrosine phosphorylation (GO:0018108), peptidyl-tyrosine modification (GO:0018212), protein autophosphorylation (GO:0046777), and cellular response to peptide hormone stimulus (GO:1901653); these results suggest that the therapeutic effect may involve intervention in protein phosphorylation, tyrosine modification, and hormone signaling pathways (Figure 1C). Notably, insulin-related processes including response to insulin (GO:0032868) and insulin receptor signaling pathway (GO:0008286) were also among the screened biological processes (*p* < 0.05). The results also showed that the targets may have functions in histone kinase activity, protein serine/threonine kinase activity, and protein tyrosine kinase activity (Figure 1D), and receptor complex, membrane raft, and membrane microdomain were the main cellular components (Figure 1E). KEGG enrichment analysis revealed that the therapeutic effects occur mainly through the MAPK signaling pathway (hsa04010), PI3K-Akt signaling pathway (hsa04151), Rap1 signaling pathway (hsa04015), calcium signaling pathway (hsa04020), and Ras signaling pathway (hsa04014) (Figure 1F).

### 3.3. 3T3-L1 Preadipocyte Differentiation

**Figure 2:**
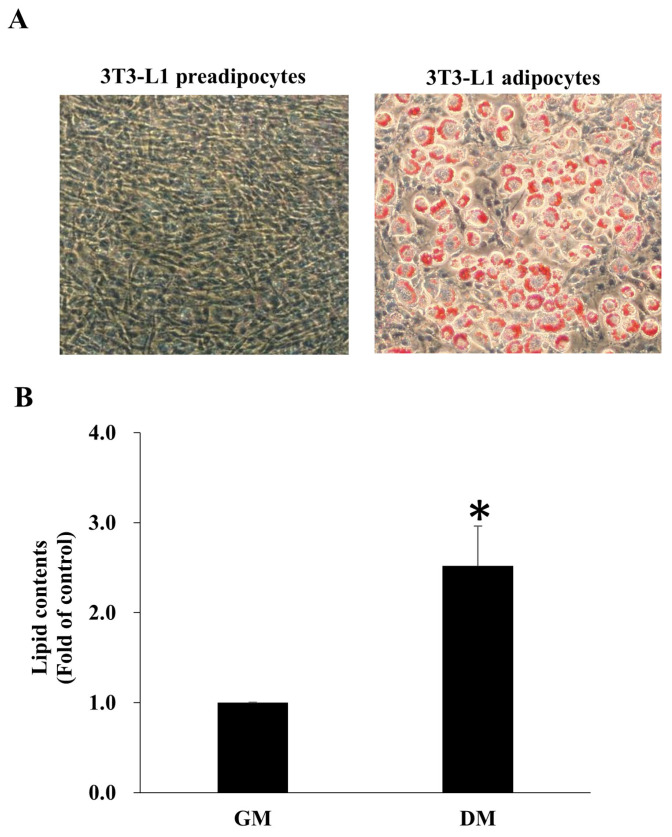
3T3-L1 preadipocyte differentiation and lipid accumulation. 3T3-L1 cells were induced to differentiate for 8 days, after which they were fixed and stained with Oil Red O. Cellular morphology was examined under a microscope, and lipid accumulation was assessed by Oil Red O staining. (**A**) Representative images from one of three independent experiments are shown (200× magnification). (**B**) Data are presented as mean ± SD from three independent experiments. * *p* < 0.05 vs. GM group. DM, differentiation medium; GM, growth medium.

To study IL-1β-induced IR in adipocytes, we first made 3T3-L1 preadipocytes differentiate to adipocytes by addition of differentiation medium I and II for 8 days. After 8 days, lipid accumulation in cells was confirmed by using Oil red O staining (Figure 2A). The lipid content was significantly greater in 3T3-L1 adipocytes than in 3T3-L1 preadipocytes (Figure 2B).

### 3.4. deAND Shows No Detrimental Effect on Cell Viability but Inhibits Insulin Resistance Induced by IL-1β

**Figure 3:**
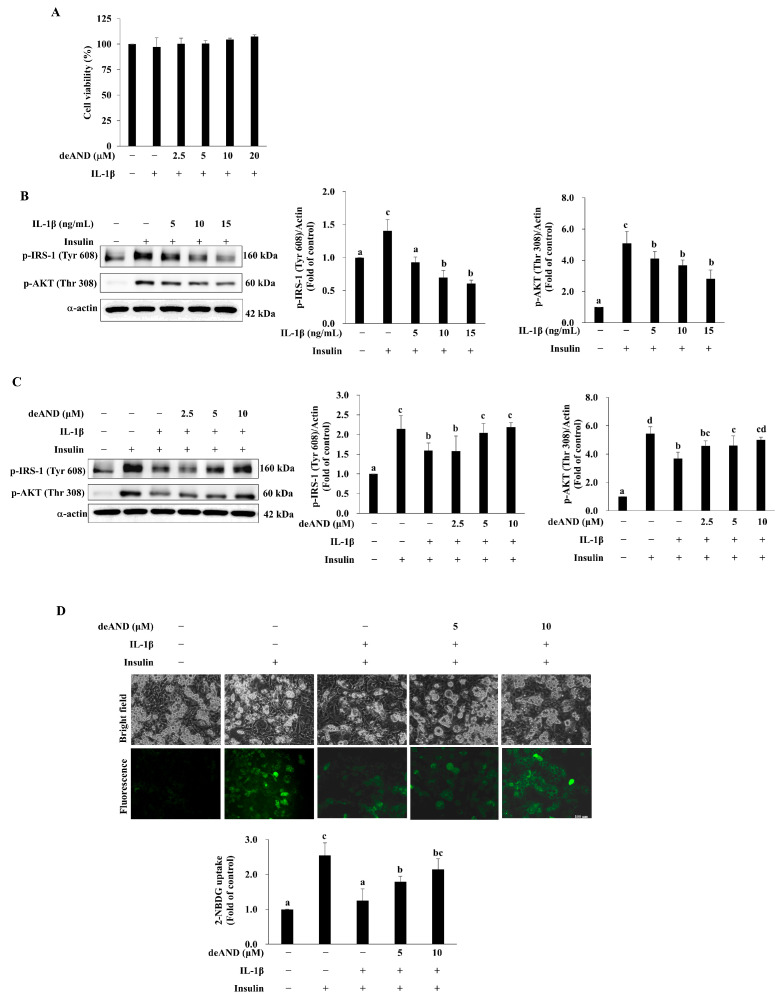
deAND does not affect cell viability but prevents IL-1β-induced insulin resistance. (**A**) Cells were pretreated with deAND (2.5, 5, 10, or 20 μM) for 16 h and then incubated with IL-1β (10 ng/mL) for an additional 24 h. Cell viability was assessed using the MTT assay. (**B**) Cells were treated with IL-1β (5, 10, or 15 ng/mL) for 24 h, followed by 100 nM insulin stimulation for 30 min. Protein levels of p-IRS-1 (Tyr608), p-AKT (Thr308), and α-actin were analyzed by Western blotting. (**C**) Cells were pretreated with deAND (2.5, 5, or 10 μM) for 16 h, and then incubated with IL-1β (10 ng/mL) for 24 h, followed by 100 nM insulin stimulation for 30 min. Protein levels of p-IRS-1 (Tyr608), p-AKT (Thr308), and α-actin were determined by Western blotting. (**D**) Cells were pretreated with deAND (5 or 10 μM) for 16 h, and then incubated with IL-1β (10 ng/mL) for 24 h, followed by 100 nM insulin in the presence of 600 nM 2-NBDG for 30 min. Intracellular 2-NBDG uptake was assessed by fluorescence microscopy (scale bar = 100 μm). Data are presented as mean ± SD of three independent experiments. Values not sharing the same letter differ significantly (*p* < 0.05).

Inflammation is an important cause of diabetes [25], and pro-inflammatory cytokines are higher in patients with diabetes than in nondiabetic persons [26]. Many studies have investigated the role of TNFα in IR using both in vivo [17,27] and in vitro [17,28] models; however, few researchers have studied the role of IL-1β in IR. Before corroborating the inhibitory effect of deAND on IL-1β-induced IR in 3T3-L1 adipocytes, we studied the effect of deAND on cell viability in the presence of IL-1β. As shown in Figure 3A, cell viability did not differ significantly among groups of cells pretreated with deAND at concentrations up to 20 μM for 16 h followed by incubation with 10 ng/mL IL-1β for an additional 24 h. However, when cells were exposed to IL-1β alone, insulin-induced phosphorylation of IRS-1 (Tyr 608) and AKT (Thr 308) was dose-dependently inhibited (Figure 3B), suggesting that IL-1β damages the insulin signaling pathway. IL-1β induced IR in 3T3-L1 adipocytes; however, pretreatment with deAND in concentrations of 5 μM and higher significantly attenuated the inhibition of insulin signaling by IL-1β (Figure 3C). Likewise, IL-1β impairment of insulin-induced glucose uptake was mitigated by pretreatment with deAND (Figure 3D).

### 3.5. deAND Attenuates IL-1β-Induced Phosphorylation of IRS-1 at Serine 307 Through Inhibition of ERK and JNK Activation in 3T3-L1 Adipocytes

**Figure 4:**
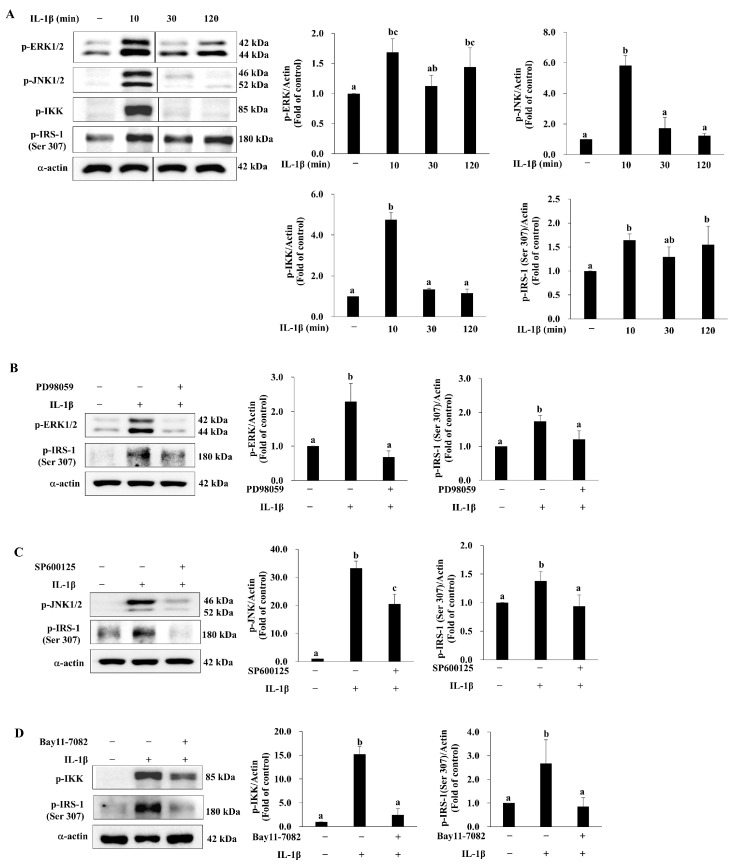
IL-1β induces IRS-1 (Ser307) phosphorylation through ERK, JNK, and IKK pathways. (**A**) Cells were treated with IL-1β (10 ng/mL) for 0, 10, 30, and 120 min. (**B**–**D**) Cells were pretreated with PD98059 (15 μM), SP600125 (10 μM), or Bay11-7082 (20 μM) for 2 h, followed by stimulation with IL-1β (10 ng/mL) for 10 min. Protein levels of p-ERK, p-JNK, p-IKK, p-IRS-1 (Ser307), and α-actin were analyzed by Western blotting. Data are expressed as mean ± SD from three independent experiments. Values not sharing the same letter are significantly different (*p* < 0.05).

It is well established that serine/threonine phosphorylation of IRS-1 is enhanced in IR and T2DM [29] through activation of the MAPKs, i.e., ERK and JNK, as well as IKKβ [30]. This is supported by the findings that constitutively active MAPK/ERK kinase (MEK) 1, MAPK kinase (MKK) 6, and MKK7 suppress tyrosine phosphorylation of insulin receptor, IRS-1, and IRS-2, as well as activation of PI3K and AKT in 3T3-L1 adipocytes in response to insulin stimulation [31]. Mutation of serine 307 to alanine prevents phosphorylation of IRS-1 by JNK and abrogates the inhibition of insulin-stimulated tyrosine phosphorylation of IRS-1 by TNFα [32]. Disruption of IKKβ activity mitigates obesity- and diet-induced IR [33]. As shown in Figure 4A, IL-1β rapidly activated ERK, JNK, and IKK after treatment for 10 min. In line with these changes, phosphorylation of IRS-1 at serine 307 by IL-1β was significant after 10 min as well. To validate the roles of MAPKs and IKK in the phosphorylation of IRS-1 at serine 307 by IL-1β, we used their respective inhibitors. As shown in Figure 4B–D, PD98059 (ERK inhibitor), SP600125 (JNK inhibitor), and Bay11-7082 (IKK inhibitor) inhibited IL-1β-induced activation of ERK, JNK, and IKK. Importantly, all inhibitors mitigated the phosphorylation of IRS-1 at serine 307 by IL-1β. These results suggest that the kinases ERK, JNK, and IKK are involved in the IL-1β-induced phosphorylation of IRS-1 at serine 307, which impairs the downstream insulin signaling pathway.

**Figure 5:**
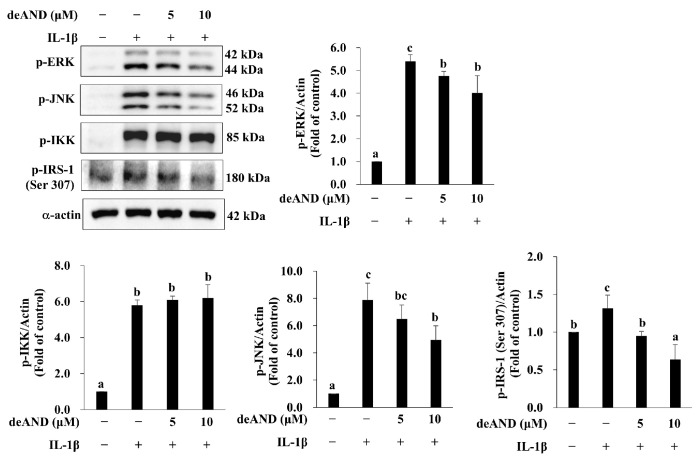
deAND suppresses IL-1β-induced IRS-1 (Ser307) phosphorylation by inhibiting ERK and JNK activation. Cells were pretreated with deAND (5 or 10 μM) for 16 h and then exposed to IL-1β (10 ng/mL) for 10 min. Protein levels of p-ERK, p-JNK, p-IKK, p-IRS-1 (Ser307), and α-actin were analyzed by Western blotting. Data are presented as mean ± SD of three independent experiments. Values not sharing the same letter differ significantly (*p* < 0.05).

Next, we investigated the upstream kinases affected by deAND to restore insulin signaling. As shown in Figure 5, IL-1β significantly phosphorylated ERK, JNK, IKK, and IRS-1 (at serine 307). However, pretreatment with deAND for 16 h mitigated the IL-1β-induced phosphorylation of ERK, JNK, and IRS-1 at serine 307 but not that of IKK, suggesting that deAND rescued insulin signaling by suppressing ERK and JNK activation.

### 3.6. deAND Attenuates IL-1β-Induced ROS Generation by Inhibiting NOX2 Complex Formation in 3T3-L1 Adipocytes

**Figure 6:**
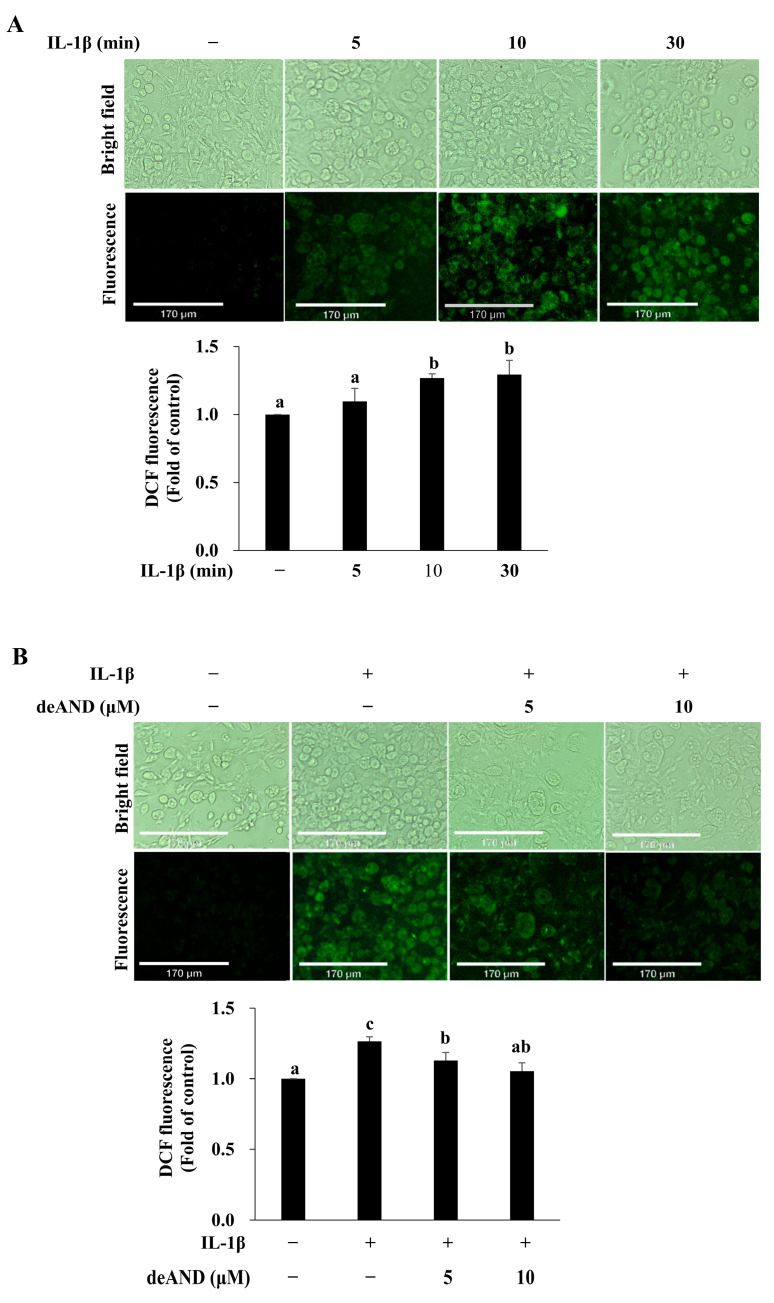
IL-1β induces ROS generation, which is attenuated by deAND pretreatment in 3T3-L1 adipocytes. (**A**) Cells were incubated with DCFDA (40 μg/mL) for 40 min and then treated with IL-1β (10 ng/mL) for 5, 10, or 30 min. Cells were fixed with 10% formalin to preserve morphology, and fluorescence intensity was assessed by fluorescence microscopy (scale bar = 170 nm). (**B**) Cells were pretreated with deAND (5 or 10 μM) for 16 h, followed by incubation with DCFDA (40 μg/mL) for 40 min and stimulation with IL-1β (10 ng/mL) for 10 min. Cells were fixed with 10% formalin, and fluorescence intensity was recorded by fluorescence microscopy. Data are expressed as mean ± SD from three independent experiments. Values not sharing the same letter are significantly different (*p* < 0.05).

ROS play an important role in the signal transduction mediating normal physiology and aberrant pathophysiology [34]. A disturbed redox balance due to excess ROS accumulation leads to the development of diseases associated with inflammatory signaling and metabolic dysfunction, such as atherosclerosis and T2DM [35]. NOX are one of the major sources of ROS in vivo. It has been reported that lack of functional NOX in monocytes/macrophages is associated with decreased plasma oxLDL, and deficiency of NOX in vascular wall cells mitigates the expression of cellular adhesion molecules in the aortas of apoE^−/−^ mice. Both events contribute to the reduced ROS production responsible for mitigating atherosclerosis development [36]. Since ROS are vital for T2DM development, we investigated the role of IL-1β in ROS generation. As shown in Figure 6A, IL-1β induced ROS generation in a time-dependent manner and a significant effect was observed after treatment for 10 and 30 min. However, when cells were pretreated with deAND (5 or 10 μM) for 16 h, the IL-1β-induced ROS generation was significantly mitigated (Figure 6B).

**Figure 7:**
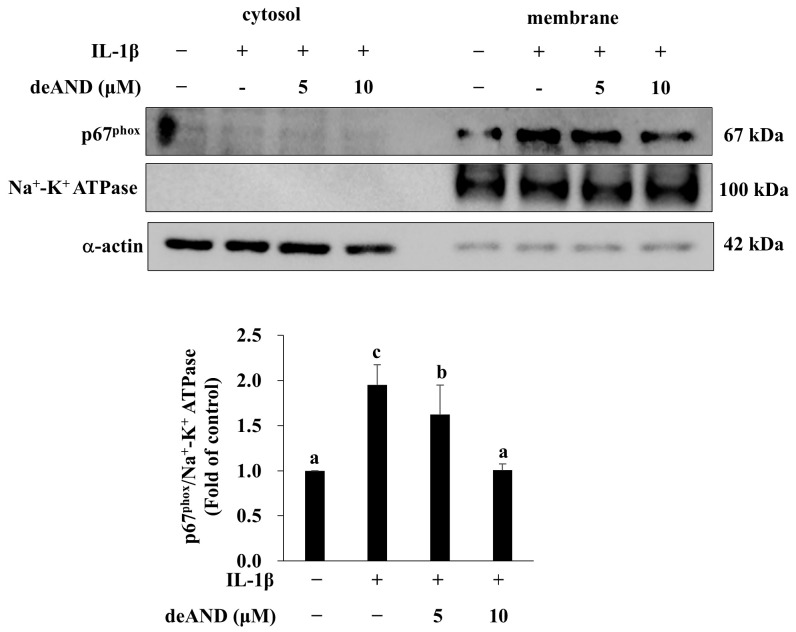
deAND inhibits NADPH oxidase 2 complex formation in 3T3-L1 adipocytes. Cells were pretreated with deAND (5 or 10 μM) for 16 h and then exposed to IL-1β (10 ng/mL) for 10 min. Protein levels of p67phox, NA+-K+ ATPase, and α-actin were analyzed by Western blotting. Data are presented as mean ± SD from three independent experiments. Values not sharing the same letter differ significantly (*p* < 0.05).

Obesity stimulates changes in T-cell subsets and increases the infiltration of activated macrophages into adipose tissue [37], and NOX activation in those recruited macrophages is responsible for the ROS overproduction [38]. A previous study showed that NOX2 knockout HFD-fed mice exhibit reduced adipose tissue inflammation compared with mice without NOX2 knockout [11]. This result suggests that NOX2 in immune cells may play a role in adipose tissue inflammation. However, whether NOX2-derived ROS play a critical role in adipocyte-related IR during the progression of obesity has not been clarified and warrants further investigation. As shown in Figure 7, IL-1β significantly induced p67phox membrane translocation, which is an indicator of NOX2 activation [39]. Furthermore, IL-1β-induced p67phox membrane translocation was dose-dependently decreased by deAND pretreatment. These results suggest that deAND pretreatment attenuated IL-1β-induced ROS generation by suppressing the activation of NOX2.

## 4. Discussion

deAND is a diterpenoid isolated from A. paniculate [18]. deAND has been documented to own various biological activities, such as anti-inflammatory, antioxidant, hepatoprotective, and hypoglycemic [16,40,41,42]. Because of these reported activities, we were interested in studying whether deAND has potential for treating IR, which is an early stage of T2DM development that is highly associated with inflammation. Using 3T3-L1 adipocytes, we studied the protective effect of deAND on IL-1β-induced IR and the underlying mechanisms involved. We found that deAND could ameliorate IL-1β-induced IR and that this effect was likely associated with the inhibition of NOX2 activation, ROS generation, ERK- and JNK-impaired insulin signaling, and consequent recovery of glucose uptake, all of which led to the reversal of inflammation-mediated IR.

Network pharmacology is an effective tool to explore the potential link between drugs, targets, and diseases [43,44]. In this study, we first explored the potential molecular mechanisms of deAND for IR through network pharmacology analysis. PPI network and GO analysis results suggested that deAND was closely related to the regulation of protein phosphorylation, tyrosine modification, and cellular response to hormone stimulus via core genes such as SRC, EGFR, AKT1, MTOR, etc. It is worth noting that KEGG analysis results showed that the targets were enriched in the MAPK signaling pathway, PI3K-Akt signaling pathway, and Ras signaling pathway, which play remarkable roles in IR progression. The MAPK pathway, particularly ERK and JNK signaling, serves as crucial signaling pathways that bridge inflammatory signals to intracellular machinery, regulating cellular processes such as insulin sensitivity and glucose metabolism [31,45]. TNFα activates three different MAPKs (ERK, p38, and JNK) and induces IR through distinct mechanisms involving IRS-1 serine phosphorylation and subsequent impairment of insulin signal transduction [31]. Our previous study demonstrated that TNFα-induced ROS generation by inducing NOX2 activation in EA.hy926 cells [16]. In addition, deAND treatment significantly reduced IRS-1 serine 307 phosphorylation while restoring IRS-1 tyrosine 608 and AKT threonine 308 phosphorylation, indicating the restoration of normal insulin signaling cascade [17]. These findings guided the research direction for our subsequent investigations into the underlying mechanisms of deAND’s antidiabetic properties.

Adipocytes express a large amount of GLUT4, which is stored in the intracellular GLUT4 storage vesicle (GSV). When adipocytes are exposed to insulin, GSV fuses with the plasma membrane by activating the insulin receptor/IRS-1/PI3K/AKT/AS160 signaling pathway [46]. Phosphorylation of IRS-1 at serine 307, however, leads to IR because of interference with insulin signal transduction [12]. Chronic inflammation is one of the risk factors for abnormal IRS-1 serine 307 phosphorylation [47]. As shown in Figure 3B, insulin-induced phosphorylation of IRS-1 (Tyr 608) and AKT (Thr 308) in 3T3-L1 adipocytes was dose-dependently inhibited by IL-1β. In addition, IL-1β significantly reduced the glucose uptake in adipocytes in response to insulin stimulation (Figure 3D). These results are consistent with those of a study performed by Jager and coworkers, who showed that IL-1β-induced IR was via a reduction in the protein expression of IRS-1 and GLUT4 membrane translocation [48]. Therefore, a sustained increase in the expression of IL-1β in adipose tissue during obesity could play a critical role in the development of IR [48].

Inflammation-induced IR is partly associated with increased and prolonged cellular production of ROS, which exposes cells to oxidative stress. Oxidative stress is documented as a risk factor for numerous inflammation-associated disorders [49]. In fact, the role of TNFα in increasing the prevalence of IR is explained by its stimulation of ROS production in adipocytes, muscle cells, and hepatocytes, which consequently damages insulin signaling by raising IRS-1 serine 307 phosphorylation [50]. Angiotensin II-induced IR in vascular smooth muscle cells also relies on ROS-driven IRS-1 phosphorylation at serine 307 and the subsequent proteasome-dependent degradation [51]. The IRS-1 serine 307 residue is near the phosphotyrosine-binding domain of IRS-1 [52]. As a result, the phosphorylation of serine 307 leads to the dissociation of IRS-1 from the Juxtamembrane domain of the insulin receptor and interrupts insulin signaling transduction [53]. A number of enzymes including NOX, inducible nitric oxide synthase, and xanthine oxidase are responsible for the generation of ROS in response to stimuli [54]. NOX are one kind of the major oxidases responsible for cellular ROS production in response to TNFα and IL-1β [55]; therefore, NOX are the link between inflammation and oxidative stress [11]. NOX2 is a complex that includes membrane components (gp91phox and p22phox) and cytosolic components (p47phox, p67phox, and p40phox) which combine at the plasma membrane to become functional [56]. Evidence indicates that NOX2 is responsible for the ROS production in bone marrow neutrophils and macrophages in mice fed a HFD, which leads to elevation of hepatic inflammation [57]. NOX2 has been shown to be expressed in adipocytes [11] and this raises the possibility that NOX2 plays an important role in ROS production under inflammatory conditions in adipocytes. As shown in Figure 6A and Figure 7, levels of ROS and membrane translocation of p67phox in adipocytes were significantly increased with IL-1β treatment. In line with these changes, increases in the activation of ERK, JNK, and IKK as well as the phosphorylation of IRS-1 at serine 307 (Figure 4A) and damage to insulin signal transduction as evidenced by decreases in phosphorylation of IRS-1 Tyr608 and AKT Thr308 (Figure 3B) were noted in cells exposed to IL-1β. These results illustrate that IL-1β acts as a stimulator of ROS production, which contributes to the onset and the progression of IR.

Because of the critical role of NOX2 in ROS production in adipocytes under inflammation, inhibiting NOX2 assembly and activation might alleviate the oxidative stress-mediated disordered condition. For instance, mice lacking NOX2 exhibit reduced size of visceral adipose deposits, attenuated hypertrophy of visceral adipocytes, and diminished infiltration of macrophages into visceral adipose tissue compared with wild-type mice [11]. We showed that deAND attenuates TNFα-induced ROS generation by inhibiting NOX2 activation in EA.hy926 cells in our previous study [16]. deAND has been shown to inhibit high-fat and high-cholesterol diet-induced increases in liver IL-1β content and to upregulate the activities of antioxidant enzymes in liver [41]. Also, deAND has been shown to improve IR in HFD-induced obese mice and to reverse the detrimental effect of TNFα on the insulin signaling pathway and glucose uptake in 3T3-L1 adipocytes [17]. Moreover, deAND is a potent agent for enhancing antioxidant defense both in vitro [16] and in vivo [41]. We were interested here in whether deAND could inhibit IL-1β-induced NOX2 activation. As shown in Figure 7, we found that membrane translocation of p67phox in response to IL-1β was suppressed by pretreatment with deAND. Consequently, cellular ROS generation was apparently mitigated (Figure 6B), suggesting that deAND likely suppresses NOX2 assembly at the plasma membrane. ROS are known to hasten IR in a kinase-dependent manner, and we showed that activation of ERK and JNK and phosphorylation of IRS-1 at serine 307 by IL-1β was reduced in cells pretreated with deAND (Figure 5). Again, IL-1β-induced defects in IRS-1 and AKT activation (Figure 3C) and glucose uptake (Figure 3D) in the presence of insulin were restored by this diterpenoid.

## 5. Conclusions

**Figure 8:**
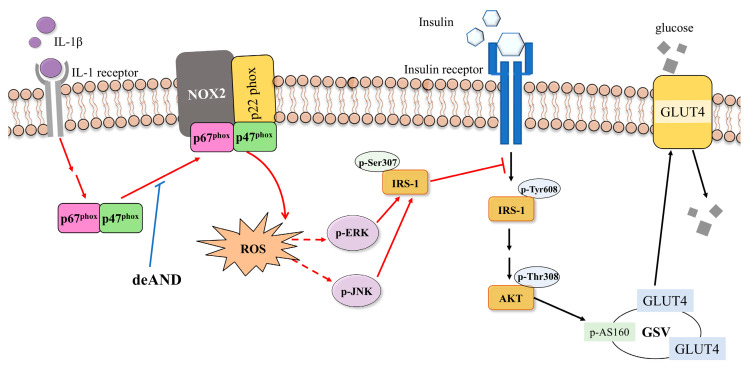
Schematic illustration of deAND-mediated inhibition of IL-1β-induced insulin resistance in 3T3-L1 adipocytes. deAND suppresses IL-1β-induced p67phox membrane translocation, NADPH oxidase 2 activation, ROS generation, and phosphorylation of ERK and JNK, thereby restoring glucose uptake. GSV, GLUT4 storage vesicle.

Our findings suggest that deAND holds promise as a potential agent with antidiabetic properties. The mechanisms through which deAND mitigates IR involve suppressing IL-1β-induced activation of NOX2, reducing ROS production, inhibiting ERK and JNK activation, and improving insulin signaling to enhance glucose uptake (Figure 8). These outcomes advocate for the potential therapeutic use of deAND in addressing IR linked to inflammation associated with obesity.

## Data Availability

The data presented in this study are available upon request from the corresponding author.

## Supplementary Materials

The following supporting information can be downloaded at: https://www.mdpi.com/article/10.3390/antiox14101155/s1. Supplementary Table S1. List of 100 proteins predicted by SwissTargetPrediction.

## Abbreviations

The following abbreviations are used in this manuscript:

IR	Insulin resistance
T2DM	Type 2 diabetes mellitus
IL-1β	Interleukin-1β
deAND	14-Deoxy-11,12-didehydroandrographolide
AND	Andrographolide
NOX2	NADPH oxidase 2
ROS	Reactive oxygen species
DMEM	Dulbecco’s Modified Eagle’s Medium
IBMX	3-Isobutyl-1-methylxanthine
APE	*A. paniculata ethanolic extract*
STRING	Search Tool for the Retrieval of Interacting Genes/Proteins
GO	Gene Ontology
KEGG	Kyoto Encyclopedia of Genes and Genomes
BP	Biological Process
MF	Molecular Function
CC	Cellular Component
LC-MS	Liquid Chromatography-Mass Spectrometry
MTT	3-(4,5-Dimethylthiazol-2-yl)-2,5-diphenyltetrazolium bromide
PBS	Phosphate-buffered saline
SDS	Sodium dodecyl sulfate
PVDF	Polyvinylidene difluoride
DCFDA	2′,7′-Dichlorodihydrofluorescein diacetate
2-NBDG	2-(N-(7-nitrobenz-2-oxa-1,3-diazol-4-yl)amino)-2-deoxyglucose
MEK	MAPK/ERK kinase
MKK	MAPK kinase
IKK	IκB kinase
PI3K	Phosphoinositide 3-kinase
PDK1	3-Phosphoinositide-dependent protein kinase-1
GLUT4	Glucose transporter 4
GSV	GLUT4 storage vesicle
HFD	High-fat diet
