# Supplementary material for: 14-Deoxy-11,12-didehydroandrographolide Alleviates IL-1β-Induced Insulin Resistance by Modulating NOX2-Driven ROS Generation and Restoring Insulin Signaling in 3T3-L1 Adipocytes
Source: Antioxidants (Basel). 2025 Sep 24;14(10):1155. doi: 10.3390/antiox14101155 (PMC12561699; doi:10.3390/antiox14101155)
Supplement: Supplementary file 1 [file antioxidants-14-01155-s001.zip › antioxidants-3816313-supplementary.pdf]

Supplementary Table S1. List of 100 proteins predicted by SwissTargetPrediction

Gene Symbol	Protein Name
Ins2	Insulin-2
Akt1	RAC-alpha serine/threonine-protein kinase
Il6	Interleukin-6
Trp53	Cellular tumor antigen p53
Tnf	Tumor necrosis factor
Vegfa	Vascular endothelial growth factor A
Mapk3	Mitogen-activated protein kinase 3
Egfr	Epidermal growth factor receptor
Egf	Epidermal growth factor
Src	Proto-oncogene tyrosine-protein kinase Src
Jak3	Tyrosine-protein kinase JAK3
Nr3c1	Glucocorticoid receptor
Hcrtr2	Hypocretin (orexin) receptor type 2
Hcrtr1	Hypocretin (orexin) receptor type 1
P2rx7	P2X purinoceptor 7
Pde7a	High affinity cAMP-specific 3',5'-cyclic phosphodiesterase 7A
Ada	Adenosine deaminase
Cdk1	Cyclin-dependent kinase 1
Map2k1	Dual specificity mitogen-activated protein kinase kinase 1
Pygl	Glycogen phosphorylase, liver form
Cd38	Cyclic ADP-ribose hydrolase
Mtor	Serine/threonine-protein kinase mTOR
Aurka	Serine/threonine-protein kinase Aurora-A
Ccr5	C-C chemokine receptor type 5
Prkch	Protein kinase C eta type
Prkcq	Protein kinase C theta type
Pde10a	High affinity cGMP-specific 3',5'-cyclic phosphodiesterase 10A
Dyrk1a	Dual specificity tyrosine-phosphorylation-regulated kinase 1A
Ptgs1	Prostaglandin G/H synthase 1 (COX-1)
Grm5	Metabotropic glutamate receptor 5
Pde4b	cAMP-specific 3',5'-cyclic phosphodiesterase 4B
Chrm2	Muscarinic acetylcholine receptor M2

Chrm1	Muscarinic acetylcholine receptor M1
Mapk9	Mitogen-activated protein kinase 9 (JNK2)
Oprm1	Mu-type opioid receptor
Alox5	Arachidonate 5-lipoxygenase
Clk1	CDC-like kinase 1
Clk3	CDC-like kinase 3
Trpm8	Transient receptor potential cation channel subfamily M member 8
Tgfbr1	Transforming growth factor beta receptor 1
Mdm2	Mouse double minute 2 homolog
Prkdc	DNA-dependent protein kinase catalytic subunit
Bace2	Beta-secretase 2
Abl1	Tyrosine-protein kinase ABL1
Drd1	Dopamine receptor D1
Akr1c21	Aldo-keto reductase family 1 member C21
Mapkapk2	MAP-kinase-activated protein kinase 2
Ca13	Carbonic anhydrase 13
Ca7	Carbonic anhydrase 7
Impdh2	Inosine-5'-monophosphate dehydrogenase 2
Zap70	Zeta-chain-associated protein kinase 70
Slc6a4	Sodium-dependent serotonin transporter
Smo	Protein smoothened
Pdgfrb	Platelet-derived growth factor receptor beta
Adora2a	Adenosine A2a receptor
Lck	Tyrosine-protein kinase Lck
Ulk3	Unc-51-like kinase 3
Stk3	Serine/threonine-protein kinase 3
Csf1r	Macrophage colony-stimulating factor 1 receptor
Fyn	Proto-oncogene tyrosine-protein kinase Fyn
Flt1	Vascular endothelial growth factor receptor 1
Raf1	RAF proto-oncogene serine/threonine-protein kinase
Kit	Mast/stem cell growth factor receptor Kit
Insr	Insulin receptor
Ret	Proto-oncogene tyrosine-protein kinase receptor Ret
Aurkb	Serine/threonine-protein kinase Aurora-B
Blk	B lymphocyte kinase
Rps6ka3	Ribosomal protein S6 kinase alpha-3

Map3k11	Mitogen-activated protein kinase kinase kinase 11
Csnk2a1	Casein kinase II subunit alpha
Lyn	Tyrosine-protein kinase Lyn
Rps6ka2	Ribosomal protein S6 kinase alpha-2
Fgfr4	Fibroblast growth factor receptor 4
Csnk2a2	Casein kinase II subunit alpha'
Slk	Serine/threonine-protein kinase SLK
Clk4	CDC-like kinase 4
Clk2	CDC-like kinase 2
Alk	Anaplastic lymphoma kinase
Fgr	Tyrosine-protein kinase Fgr
Btk	Bruton tyrosine kinase
Tie1	Tyrosine-protein kinase receptor Tie-1
Tbk1	Serine/threonine-protein kinase TBK1
Ripk1	Receptor-interacting serine/threonine-protein kinase 1
Ephx2	Epoxide hydrolase 2
Nampt	Nicotinamide phosphoribosyltransferase
Comt	Catechol O-methyltransferase
Oprd1	Delta opioid receptor
Hrh1	Histamine H1 receptor
Grm1	Metabotropic glutamate receptor 1
Maob	Monoamine oxidase B
Alox5ap	Arachidonate 5-lipoxygenase activating protein
Dpp4	Dipeptidyl peptidase-4
Ccr1	C-C chemokine receptor type 1
Braf	Serine/threonine-protein kinase B-raf
Ptger1	Prostaglandin E2 receptor EP1 subtype
Taar1	Trace amine-associated receptor 1
Wnt3a	Protein Wnt-3a
Tnks2	Tankyrase-2
Mif	Macrophage migration inhibitory factor
Mapk11	Mitogen-activated protein kinase 11
Ednra	Endothelin receptor type A
Adora2b	Adenosine A2b receptor
Npy5r	Neuropeptide Y receptor type 5
Ace	Angiotensin-converting enzyme
F2	Coagulation factor II (thrombin)

Gck	Glucokinase
Gsk3a	Glycogen synthase kinase-3 alpha
Ephx1	Epoxide hydrolase 1
Hrh3	Histamine H3 receptor
Bche	Butyrylcholinesterase
